# Budget impact analysis of energy and nutrient-dense oral nutritional formulas for hospitalized pediatric disease-related malnutrition in the Middle East

**DOI:** 10.3389/fnut.2026.1824690

**Published:** 2026-06-15

**Authors:** Ghada Zaki, Wajeeh Al-Dekhail, Mohamed Mezied, Sanaa Shaaban, Yasmin El Gendy, Ashraf Osman, Khalid Saraf, Massouma Jassmi, Mahmoud Alsoufi, Ahmed Abdelmawla

**Affiliations:** 1 Institute of Global Health and Human Ecology, The American University in Cairo, Cairo, Egypt; 2 King Faisal Specialist Hospital & Research Centre, Riyadh, Saudi Arabia; 3 Dr. Sulaiman Al-Habib Medical Group, Riyadh, Saudi Arabia; 4 Department of Pediatrics and Clinical Nutrition, Ain Shams University, Cairo, Egypt; 5 Department of Pediatrics, Dar Al Shifa Hospital, Kuwait, Kuwait; 6 Nutrition Clinical Support Services, Al Jalila Children & Specialty Hospital, Dubai, United Arab Emirates; 7 Department of Cardiology, Al Jalila Children & Specialty Hospital, Dubai, United Arab Emirates; 8 Nutricia, Medical Affairs and Market Access, Dubai, United Arab Emirates

**Keywords:** budget impact analysis, disease-related malnutrition, DRM prevalence, energy and nutrient-dense formulas, hospital length of stay, Middle East, pediatric nutrition

## Abstract

**Introduction:**

Disease-related malnutrition is a significant yet underappreciated burden among hospitalized children, particularly in the Middle East, where data on prevalence and economic effects are few. DRM perpetuates a vicious cycle in which diseases lead to malnutrition, and malnutrition in turn worsens disease outcomes, prolongs recovery, and raises healthcare expenses. Although nutritional treatments are clinically useful in DRM management, information on their economic effects is sparse.

**Objective:**

To estimate the budget impact and potential cost savings of introducing energy-and nutrient-dense formulas (ENDFs) for hospitalized pediatric DRM patients aged 0–5 years across public and private sectors in Egypt, Saudi Arabia, Kuwait, and the United Arab Emirates.

**Methods:**

An Excel-based cost calculator model compared standard nutritional formulas to ENDFs over a one-year horizon from a healthcare payer’s perspective across both public and private sectors. The model estimated annual direct medical costs, incorporating population inputs, formulas, and hospitalization costs, as well as hospital length of stay (LOS), informed by expert consultations. One-way sensitivity analyses (±20%) were conducted to assess the model’s robustness. Further, Scenario analyses using alternative uptake assumptions (25, 50, and 75%) were also conducted to improve the practical relevance of the model.

**Results:**

The use of ENDFs was linked to consistent reductions in LOS (19–50%) and significant cost savings (15–50%), mainly driven by lower hospitalization expenses, which made up over 95% of total costs. Sensitivity analysis showed that prevalence, LOS reduction percentage, and baseline LOS were the main cost drivers, in line with previous economic models.

**Conclusion:**

The findings demonstrate the clinical and economic value of ENDFs in managing pediatric DRM, through reduced hospital length of stay and associated costs, supporting their broader adoption.

## Highlights

- *Evidence gap*: Disease-related malnutrition (DRM) is a prevalent yet under recognized comorbidity in hospitalized children. Despite its burden, data on the prevalence and economic impact of pediatric DRM in the Middle East are scarce. This study provides the first multi-country budget impact analysis of energy- and nutrient-dense formulas (ENDFs) across four Middle Eastern Arab countries.
- *Key findings*: ENDFs were associated with shorter hospital stays, faster recovery, and substantial cost savings driven by reduced hospitalization cost compared with standard nutritional formulas.
- *Implications*: Findings highlight the intrinsic link between nutritional intervention and economic outcomes, improved nutrition leads to faster recovery and reduced costs, supporting ENDFs as a value-based investment for healthcare systems in the Middle East and broader.

## Background

Malnutrition remains one of the most urgent global health issues, especially among infants and children, impairing their growth, development, and overall potential, while also imposing significant economic burdens on societies (1–3). Based on its etiology, malnutrition is broadly divided into two categories: non-disease-related (community) and disease-related (DRM) (2, 3). Community malnutrition is mainly caused by socioeconomic factors such as poverty, limited access to food, and poor food quality, often resulting in children experiencing stunting, wasting, or being underweight (2, 3). In contrast, DRM arises as a secondary consequence of acute or chronic diseases and is driven by disease-induced factors, including systemic inflammation, increased metabolic demands and nutrient requirements, reduced appetite, impaired swallowing, gastrointestinal dysfunction, and catabolic stress (1, 4). It is characterized by inadequate intake, absorption, or utilization of nutrients during illness, leading to weight and muscle loss, impaired functional capacity, and worsened clinical outcomes (5). DRM creates a vicious cycle where disease can trigger malnutrition, which in turn exacerbates disease progression and clinical outcomes by weakening immunity, delaying recovery, increasing complication rates, and raising morbidity and mortality (6).

Globally, DRM is one of the most common comorbidities in healthcare, especially among hospitalized pediatric patients with acute or chronic illnesses (7, 8). Reported global prevalence in this group varies widely, with estimates from 2.5 to 60% (5–9), depending on the underlying conditions, geographical regions, healthcare settings, age groups studied, and nutritional assessment methods (5, 10). It frequently co-occurs with various conditions, including infectious diseases, gastrointestinal disorders, congenital heart disease, respiratory conditions, chronic kidney and liver diseases, endocrine and metabolic disorders, and cystic fibrosis (3, 7, 11, 12).

The economic burden of DRM is substantial, driven by its negative impact on health outcomes, necessitating increased healthcare resource utilization and associated costs (1–4, 7, 10, 12). Numerous studies have linked DRM to longer hospital stays, higher readmission rates, increased demand for intensive care, and a greater risk of complications, including infections and other disease-related complications (1–4, 7–12). For instance, hospital stays are reported to be approximately 45% longer in malnourished pediatric patients compared to well-nourished patients (11). Furthermore, a cost-of-illness study showed a 40% increase in hospital expenditures for affected pediatric patients compared to those without malnutrition (3).

Evidence suggests that timely nutritional support, including oral nutritional supplements (ONS) and enteral tube feeding (ETF), is an essential component of care for patients with DRM, contributing to improved clinical outcomes and reduced healthcare costs (11, 13). Among nutritional interventions, energy and nutrient-dense formulas (ENDF) have demonstrated particular benefits for infants and children with DRM and growth failure (14–17). Studies report that ENDFs are well tolerated and support improved energy and nitrogen balance, weight gain, and overall nutrient intake, promoting catch-up growth and recovery (14–17). In a randomized controlled trial involving infants with congenital heart disease post-surgery, ENDFs use was associated with shorter hospital stays and reduced antibiotic use compared to standard formulas (14).

Despite the recognized benefits of nutritional interventions for managing pediatric DRM, global evidence regarding their health-related economic outcomes remains limited, particularly in the Middle East, where data on prevalence, clinical burden, and healthcare costs are scarce. To address these gaps, this study conducts the first multi-country budget impact analysis of ENDFs for pediatric DRM management across four Middle Eastern countries. The findings from this analysis provide critical economic evidence to inform healthcare decision-making and resource allocation both within and beyond the region.

### Study objective and scope

This study aims to estimate the budgetary impact of introducing energy and nutrient-dense formulas (ENDFs), specifically Infatrini™ and Nutrini™, for the nutritional management of hospitalized pediatric patients aged 0–5 years with DRM in four Middle Eastern countries [Egypt, Saudi Arabia (KSA)], Kuwait, and the United Arab Emirates (UAE). The analysis focuses on the potential annual cost savings driven by clinical improvements, particularly hospital length of stay reductions, compared to standard formulas currently administered in hospital settings. To our knowledge, this is the first study to evaluate the economic impact of ENDFs for pediatric DRM in the Middle East. Additionally, the study provides region-specific data on the prevalence and associated clinical outcomes, particularly hospital length of stay.

## Methods

### Study perspective and time horizon

The analysis was conducted from the perspective of healthcare payers, considering both public and private healthcare sectors across the four target countries. The model employed a 1-year time horizon and included only direct medical costs related to hospitalization and nutritional interventions. All costs were reported in local currency, i.e., Saudi Riyal (SAR) for KSA, Kuwaiti Dinar (KWD) for Kuwait, United Arab Emirates Dirham (AED) for UAE, and Egyptian Pound (EGP) for Egypt.

### Model structure

An Excel-based cost-calculator model was used to calculate total costs and assess the budgetary impact for comparing two nutritional scenarios: (1) a standard care scenario (Isocaloric Formulas), where all hospitalized pediatric DRM patients received conventional nutritional formulas, and (2) an intervention scenario where all DRM patients received ENDFs (Infatrini™/Nutrini™) according to the age group, Infatrini™ for infants (0–1 years) and Nutrini™ for children (1–5 years).

### Model inputs

An exploratory literature review was initially conducted to collect epidemiological and clinical data on pediatric DRM across the four target Middle Eastern countries. The search utilized electronic databases, primarily Google Scholar and PubMed, alongside regional sources and international platforms such as the WHO and UNICEF websites. The review confirmed a high prevalence of community malnutrition in the Middle East and North Africa (MENA) region, affecting approximately 24 million children under five years of age. Of these, 18% are experiencing stunting (about 10 million), and 6.3% are suffering from wasting (around 3.5 million) (18). These findings align with other reports, which indicate stunting rates of up to 20% in Egypt, 12% in Saudi Arabia, and 6.9% in Kuwait (19). While these visible forms of malnutrition have been the focus of numerous global and regional studies, information specifically related to pediatric DRM remains extremely limited.

To address these data gaps, key opinion leader (KOL) interviews and expert elicitation were conducted. A total of 24 KOLs, including nutritionists, pediatricians, and payers, were consulted across eight public and private hospitals. Their insights ensured the model accurately reflected current healthcare settings, incorporating real-world clinical practices, institutional experience, and local healthcare resource utilization. Expert elicitation using structured discussions were conducted at the hospital level for each country-sector model, where three KOLs contributed to a single reported response. Following data collection, an internal data review was conducted to assess the consistency of expert inputs across settings. Where unusual or inconsistent values were identified, these were discussed with the respective KOLs for clarification and confirmation to ensure alignment with local clinical practice. A summary of the base case model structure is presented in Table 1.

**Table 1:** Base case setting of the model.

Setting	Input
Method	Expert elicitation with 24 key opinion leaders (KOLs)
Analytical tool	Excel Based Cost Calculator Model
Perspective	Public and private settings in: Egypt, Kingdom of Saudi Arabia, Kuwait, United Arab Emirates
Currency	EGP (Egypt), SAR (Saudi Arabia), KWD (Kuwait), AED (United Arab Emirates)
Eligible population	Hospitalized infants (0–1) with DRM Hospitalized children (1–5) with DRM
Comparator	Standard Formula (*Isocaloric Formula*) determined in hospital settings
Intervention	Energy Nutrient Dense Formula (ENDFs) Infatrini™ (0–1 Years) Nutrini™ (1–5 Years)
Main input source	Cross-sectional expert survey (expert elicitation) involving 24 KOLs across 8 hospitals in the four countries.
Model inputs	Population inputs
	Total annual Hospitalization in pediatric Wards % prevalence of DRM in hospitalized pediatric patients (0–1 years)(1–5 years) Estimated number of pediatric patients with DRM % further need ICU admission
	Hospital utilization resources
	LOS Ward standard LOS Ward ENDFs LOS ICU standard LOS ICU ENDFs
	Costs inputs
	Hospitalization costs Ward cost per day per patient ICU cost per day per patient Nutrition Costs per patient for standard versus ENDFs
Calculated clinical outcomes	% reduction in Length of Stay (Ward) % reduction in Length of Stay (ICU)
Calculated costs outcomes	Total Costs for standard Total Costs For Intervention Incremental cost saving between standard and ENDFs % Cost Savings

KOLs, Key opinion Leaders; LOS, Length of stay.

### Populations inputs

The model population consisted of hospitalized pediatric DRM patients aged 0–5 years, divided into two age groups: infants (0–1 years) and children (1–5 years). These groups correspond to age-appropriate ENDFs (Infatrini™/Nutrini™). Population inputs were derived from KOL estimates specific to each country and healthcare setting. Experts provided data on the annual number of pediatric inpatient admissions by age group, the proportion of patients exhibiting DRM symptoms, and the percentage requiring ICU care. These inputs defined the eligible patient population for both the standard care and nutritional intervention scenarios, ensuring that the model’s population parameters accurately reflected real-world clinical conditions and practices.

### Hospital resources utilization

The average hospital length of stay (LOS) for pediatric DRM patients receiving standard formula, Infatrini™ (0–1 year group), or Nutrini™ (1–5 year group) was collected separately for general pediatric wards and ICU settings, based on expert clinical inputs. This data was used to calculate the percentage reduction in LOS associated with the nutritional intervention, compared to standard formula use. Experts also reviewed and validated the literature findings regarding LOS reductions due to nutritional interventions in both ward and ICU settings. The resulting percentage reduction in LOS, derived from expert estimates, served as the primary clinical outcome in the model and was incorporated as a key input in the one-way sensitivity analysis, ensuring the robustness of the model’s results. This process accounted for real-world variability across healthcare settings and countries, offering a more realistic assessment of the intervention’s impact.

### Cost inputs

The model’s cost inputs included average daily hospitalization costs for pediatric wards and ICU settings, as well as the nutritional costs based on the required number of bottles per day for standard formula, Infatrini (0–1 years), and Nutrini (1–5 years). Expert input and local market prices were used to determine the cost of each formula.

### Key assumptions

- The model assumed that 100% of eligible patients received standard formulas, while 100% received Infatrini/Nutrini in the intervention scenario. The costs and outcomes for each scenario were then estimated and compared.
- Hospitalization costs were calculated based on average daily costs for both pediatric wards and ICU settings. Disease-specific medication costs were excluded, as the focus of this study was solely on the budgetary impact of the nutritional intervention.
- Reduction in hospital LOS was considered the primary indicator of clinical improvement resulting from the nutritional intervention. This assumption, based on evidence from the literature and expert clinical input, was incorporated into the model as a key driver for estimating potential cost savings associated with improved patient outcomes.
- All patients were assumed to be initially admitted to general pediatric wards, with a proportion subsequently requiring transfer to the intensive care unit (ICU), based on expert clinical input.
- The model was assumed and calculated based on a single hospital admission per patient.

### Sensitivity analysis

To test the robustness of the model outcomes against variations in input values, a one-way sensitivity analysis was performed. For this, key input values were varied by 20% from their base-case values and displayed in tornado diagrams to visually represent the sensitivity of the model to changes in each parameter.

### Scenario analysis

To improve the practical relevance of the model, scenario analyses were conducted under different uptake assumptions for Nutrini™ and Infatrini™. Partial uptake levels of 25, 50, and 75% (with the remaining patients receiving standard formula) were evaluated in addition to the original 100% uptake base-case scenario. Total costs and cost savings were compared with the standard formula baseline scenario.

## Results

Full results are detailed in Tables 2, 3 (public sector: Table 2 for infants, Table 3 for children) and Tables 4, 5 (private sector: Table 4 for infants, Table 5 for children).

**Table 2:** Public hospitals base case (age 1–5 years).

Variable	Egypt	KSA	Kuwait	UAE
Population inputs				
Hospitalizations/Year in pediatric wards	6,217	1825	2,180	2,555
DRM prevalence (%)	23.00%	50.00%	21.30%	68.00%
Number of DRM pediatric patients (1–5 Years)	1,430	913	464	1737
% of Patients further admitted to ICU	14.00%	25.00%	3.00%	22%
Hospital resources utilization				
Ward LOS SF	7.67	4.00	8.67	17.50
Ward LOS Nutrini™	4.00	2.00	5.00	9.00
ICU LOS SF	8.67	3.00	6.00	10.00
ICU LOS Nutrini™	5.67	2.00	4.00	5.00
Clinical outcomes				
% Reduction Ward LOS	47.85%	50.00%	42.33%	48.57%
% Reduction ICU LOS	34.60%	33.33%	33.33%	50.00%
Cost inputs				
Ward cost/Day/Patient	750	1,250	40	3,250
ICU cost/Day/Patient	3,000	2000	50	5,850
Nutrition costs/day/patient for SF	88.08	15.08	1.68	33.65
Nutrition costs/day/patient for Nutrini™	170.35	27.52	2.99	26.50
Final costs outcomes				
Total hospitalization costs-SF	13,432,432	5,931,250	165,212	121,174,963
Total hospitalization costs-Nutrini™	7,694,918	3,193,750	95,654	61,999,119
Total nutrition costs –SF	1,118,883	65,362	6,904	1,151,731
Total nutrition costs –Nutrini™	1,167,699	62,780	7,108	465,015
Total costs – SF	14,551,315	5,996,612	172,116	122,326,694
Total costs – Nutrini™	8,862,616	3,256,530	102,763	62,464,134
Incremental cost savings	−5,688,699	−2,740,082	−69,353	−59,862,560
% Savings	39%	46%	40%	49%

DRM, disease-related malnutrition; LOS, length of stay; SF, standard formula.

Input values represent average estimates reported by three KOLs per hospital following a structured group discussion. Individual responses were not recorded separately; therefore, measures of variability (e.g., range or standard deviation) could not be calculated.

**Table 3:** Public hospitals base case (age 0–1 years).

Variable	Egypt	KSA	Kuwait	UAE
Population inputs				
Hospitalizations/Year in pediatric wards	5,225	1825	1,300	3,650
DRM prevalence (%)	25%	50%	26.30%	61.00%
Number of DRM pediatric patients (0–1 Years)	1,306	913	342	2,227
% of Patients further admitted to ICU	18.00%	25.00%	9.00%	27%
Hospital resources utilization				
Ward LOS SF	7.67	4.00	7.00	16.00
Ward LOS Infatrini™	5.68	2.00	5.01	8.00
ICU LOS SF	8.00	3.00	7.00	8.00
ICU LOS Infatrini™	6.32	2.01	5.01	4.00
Clinical outcomes				
% Reduction ward LOS	26.00%	50.00%	28.50%	50.00%
% Reduction ICU LOS	21.00%	33.00%	28.50%	50.00%
Cost inputs				
Ward cost/Day/Patient	750	1,250	40	3,250
ICU cost/Day/Patient	3,000	2000	50	5,850
Nutrition costs/day/patient for SF	37.78	6.61	1.05	24.43
Nutrition costs/day/patient for Infatrini™	93.52	20	2.9	36.98
Final costs outcomes				
Total hospitalization costs-SF	13,157,203	5,931,250	106,502	143,912,054
Total hospitalization costs-Infatrini™	10,018,480	3,198,313	76,149	71,956,027
Total nutrition costs –SF	449,580	28,650	2,739	987,784
Total nutrition costs –Nutrini™	832,328	45,671	5,409	747,611
Total costs – SF	13,606,783	5,959,900	109,241	144,899,838
Total costs – Infatrini™	10,850,809	3,243,983	81,558	72,703,638
Incremental cost savings	−2,755,974	−2,715,917	−27,683	−72,196,200
% Savings	20%	46%	25%	50%

**Table 4:** Private hospitals base case (age 1–5 years).

Variable	Egypt	KSA	Kuwait	UAE
Population inputs				
Hospitalizations/Year in pediatric wards	1,200	1,610	425	2356.25
DRM prevalence (%)	4%	13%	5.00%	9.30%
Number of DRM pediatric patients (1–5 Years)	48	209	21	219
% of Patients further admitted to ICU	30.00%	13.00%	NA	3%
Hospital resources utilization				
Ward LOS SF	8.00	6.00	7.67	3.50
Ward LOS Nutrini™	5.00	3.67	5.67	2.50
ICU LOS SF	7.50	4.33	NA	4.50
ICU LOS Nutrini™	4.50	3.50	NA	3.50
Clinical outcomes				
% Reduction ward LOS	37.50%	38.83%	26.07%	28.57%
% Reduction ICU LOS	40.00%	19.17%	0.00%	22.22%
Cost inputs				
Ward Cost/Day/Patient	7,333	2,250	250	2000
ICU Cost/Day/Patient	10,667	4,750	0	5,000
Nutrition costs/day/patient for SF	116.181	13.99	2.13	21.34
Nutrition costs/day/patient for Nutrini™	214.31	19.9	1.9	19.39
Final costs outcomes				
Total hospitalization costs-SF	3,967,908	3,385,171	40,747	1,681,832
Total hospitalization costs-Nutrini™	2,451,142	2,180,644	30,124	1,210,700
Total nutrition costs –SF	57,161	19,217	347	16,998
Total nutrition costs –Nutrini™	65,322	17,181	229	11,069
Total costs – SF	4,025,069	3,404,388	41,094	1,698,831
Total costs – Nutrini™	2,516,463	2,197,825	30,353	1,221,769
Incremental cost savings	−1,508,606	−1,206,563	−10,741	−477,062
% Savings	37%	35%	26%	28%

**Table 5:** Private hospitals base case (age 0–1 years).

Variable	Egypt	KSA	Kuwait	UAE
Population inputs				
Hospitalizations/Year in pediatric wards	1,200	1,163	883	1,145
DRM prevalence (%)	5.00%	13.00%	8.00%	4.30%
Number of DRM pediatric patients (0–1 Years)	60	151	71	49
% of Patients further admitted to ICU	30.00%	18.00%	21.00%	3%
Hospital resources utilization				
Ward LOS SF	9.00	6.00	7.00	3.00
Ward LOS Infatrini™	6.00	6.00	4.97	2.01
ICU LOS SF	8.00	4.33	0.00	6.00
ICU LOS Infatrini™	4.80	1.21	0	4.02
Clinical outputs				
% Reduction Ward LOS	33.00%	0.00%*	29.00%	33.00%
% Reduction ICU LOS	40.00%	72.00%*	0.00%	33.00%
Cost inputs				
Ward cost/Day/Patient	7,333	2,250	250	2000
ICU cost/Day/Patient	10,667	4,750	0	5,000
Nutrition costs/day/patient for SF	96.55	11.31	1.06	19.31
Nutrition costs/day/patient for Infatrini™	149.56	29.29	2.42	36.18
Final costs outcomes				
Total hospitalization costs-SF	5,495,868	2,600,793	123,620	339,722
Total hospitalization costs-Infatrini™	3,574,708	2,197,789	87,770	227,613
Total nutrition costs -SF	66,040	11,592	524	3,023
Total nutrition costs -Infatrini™	67,033	27,537	850	3,795
Total costs – SF	5,561,908	2,612,386	124,144	342,745
Total costs- Infatrini™	3,641,741	2,225,325	88,620	231,409
Incremental cost savings	−1,920,167	−387,060	−35,524	−111,336
% Savings	35%	15%	29%	32%

*This finding (0% ward LOS reduction, 72% ICU LOS reduction) was identified as unusual during internal data review. The KOLs were re-contacted and re-confirmed their original estimates, explaining that in their clinical experience, ward length of stay showed no reduction while ICU length of stay showed substantial reduction. The inputs were retained as reported.

### Clinical outcomes

Reduction in hospital length of stay (LOS) between standard formulas and ENDFs was the primary clinical outcome and a key indicator of clinical improvement in this study. Across all countries, healthcare settings, and both pediatric age groups (0–1 and 1–5 years), the utilization of Infatrini™ and Nutrini™ led to consistent LOS reductions in both general wards and ICUs.

### Public hospitals

In Egypt, LOS decreased by 26% in wards and 21% in ICUs among infants, and by 48% in wards and 35% in ICUs among children. In Kuwait, reductions were 25.5% (ward) and 28.5% (ICU) for infants, and 42% (ward) and 33% (ICU) for children. In KSA, both age groups experienced LOS reductions of 50% in wards and 33% in ICUs. The UAE demonstrated the greatest overall reductions, with LOS reduced by 50% across both care settings and age groups.

### Private hospitals

In the private sector, LOS reductions were observed across most countries and age groups, though generally less pronounced than in the public sector. In Egypt, LOS declined by 33% (ward) and 40% (ICU) among infants, and 38% (ward) and 40% (ICU) among children. In Kuwait, reductions were seen among infants (29% in wards), with no ICU data available; children experienced reductions of 26% (ward) and 33% (ICU). In KSA, infants showed a marked ICU reduction of 72%, with no change in ward LOS; for children, LOS declined by 39% in wards and 19% in ICUs. In the UAE, reductions were 33% (ward) and 22% (ICU) among infants, and 29% (ward) and 22% (ICU) among children.

### Economic outcomes

The results demonstrated consistent net cost savings for ENDFS across all models evaluated. These savings were primarily driven by reductions in hospital length of stay (LOS) and the associated decrease in hospitalization costs.

### Public hospitals

Across public hospitals in all countries, percentage cost reductions ranged from 20 to 50%. The UAE showed the highest % cost savings, with Nutrini™ reducing costs by AED 59.86 million, representing a 49% reduction, and Infatrini™ by AED 72.2 million, a 50% reduction. In Kuwait, Nutrini™ led to annual savings of KWD 69,353 (40% reduction), while Infatrini™ generated KWD 27,683 (25% reduction). In KSA, both Nutrini™ and Infatrini™ showed comparable savings of SAR 2.7 million (46% reduction). In Egypt, the introduction of Nutrini™ among children resulted in annual savings of EGP 5.69 million, accounting for a 39% reduction for children and EGP 2.76 million, accounting for a 20% reduction for infants using Infatrini™.

### Private hospitals

In most settings, the UAE, KSA, and Kuwait, the public sector demonstrated higher cost savings than the private sector for both Nutrini and Infatrini. However, Egypt showed comparable cost savings between the public and private sectors for Nutrini™, and a higher percentage reduction for Infatrini™ in the private sector.

The highest percentage savings in private hospitals were observed in Egypt, where Nutrini™ showed annual savings reached EGP 1.51 million, accounting for a 37% reduction, while Infatrini™ showed EGP 1.91 million, accounting for a 34% reduction. In KSA, savings were SAR 1.21 million (35%) for Nutrini™ and SAR 0.40 million (15%) for Infatrini™. Kuwait recorded savings of KWD 10,741 (26%) for Nutrini™ and KWD 36,236 (29%) for Infatrini™. The UAE reported AED 0.48 million (28%) for Nutrini™ and AED 0.11 million (33%) for Infatrini™.

### Costs segmentation

As shown in the cost outcomes section of Tables 2–5, across all studied countries and settings, hospitalization costs accounted for the vast majority of total healthcare expenditures, consistently exceeding 90% in both standard and ENDFs scenarios. Nutrition-related costs accounted for a small proportion of total expenditures and were modestly higher under intervention scenarios (ENDFs; Infatrini™, or Nutrini™) due to the higher unit cost of these specialized formulas. However, these increases were consistently offset by reductions in hospital length of stay and associated hospitalization costs, resulting in robust and positive net savings across all modeled settings.

### Sensitivity analysis results

**Figure 1:**
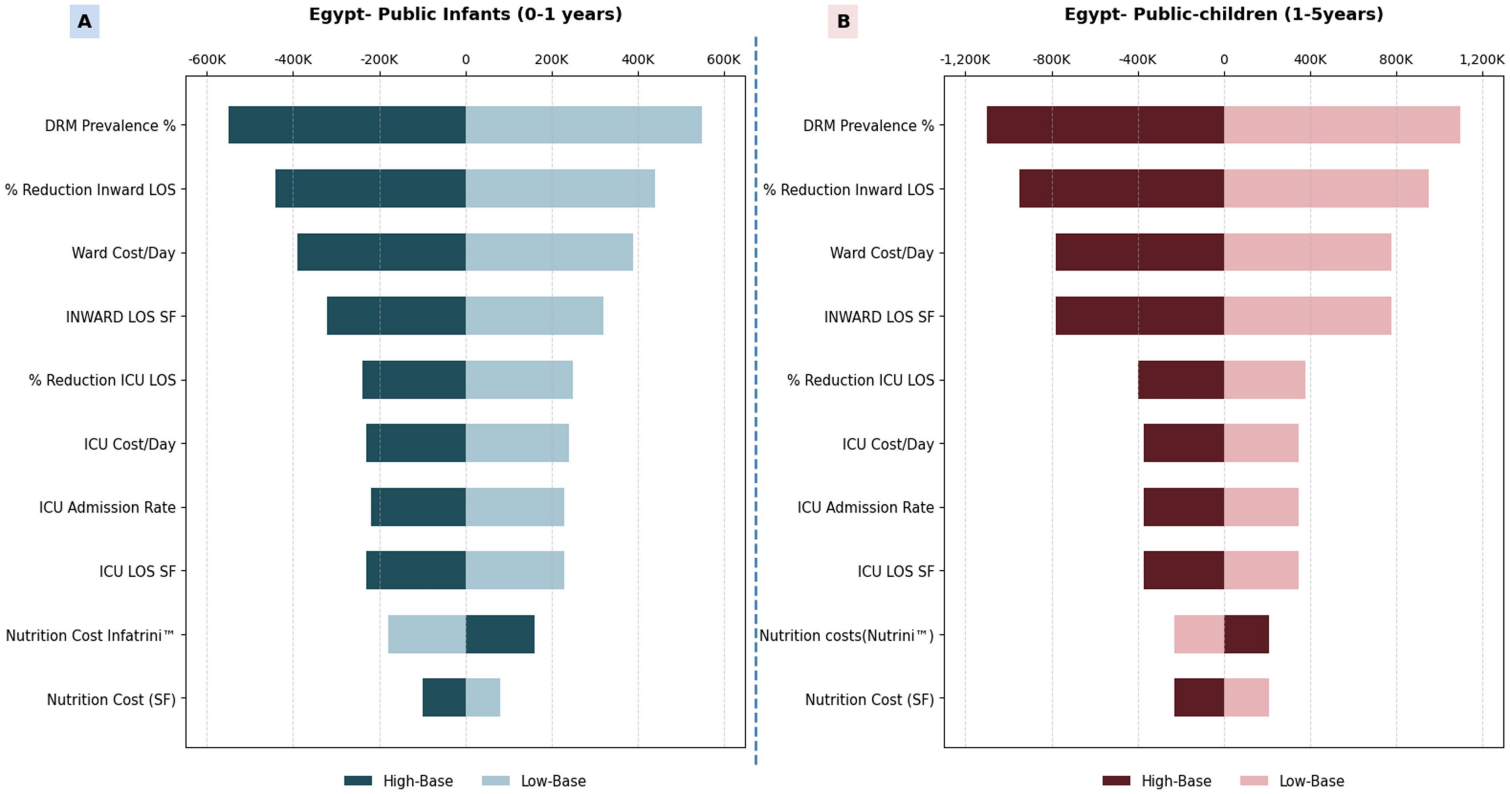
One-way sensitivity analysis tornado diagrams for the public sector. **(A)** The tornado diagram for infants (0–1 years) in Egypt, modeled for Infatrini™ versus standard formula (SF). **(B)** Children (1–5 years) in Egypt, modeled for Nutrini™ versus SF. The cost differences are expressed in Egyptian pounds currency, local currency units. All parameters were varied ±20%. Across all panels, blue shades (dark and light) indicate infants (0–1 years, Infatrini™), while orange shades indicate children (1–5 years, Nutrini™). For each parameter, the darker shade represents the impact on cost savings when the parameter is increased (+20%), while the lighter shade shows the impact when decrease (−20%). Bars represent the impact of varying key parameters on cost savings, with longer bars indicating greater sensitivity.

**Figure 2:**
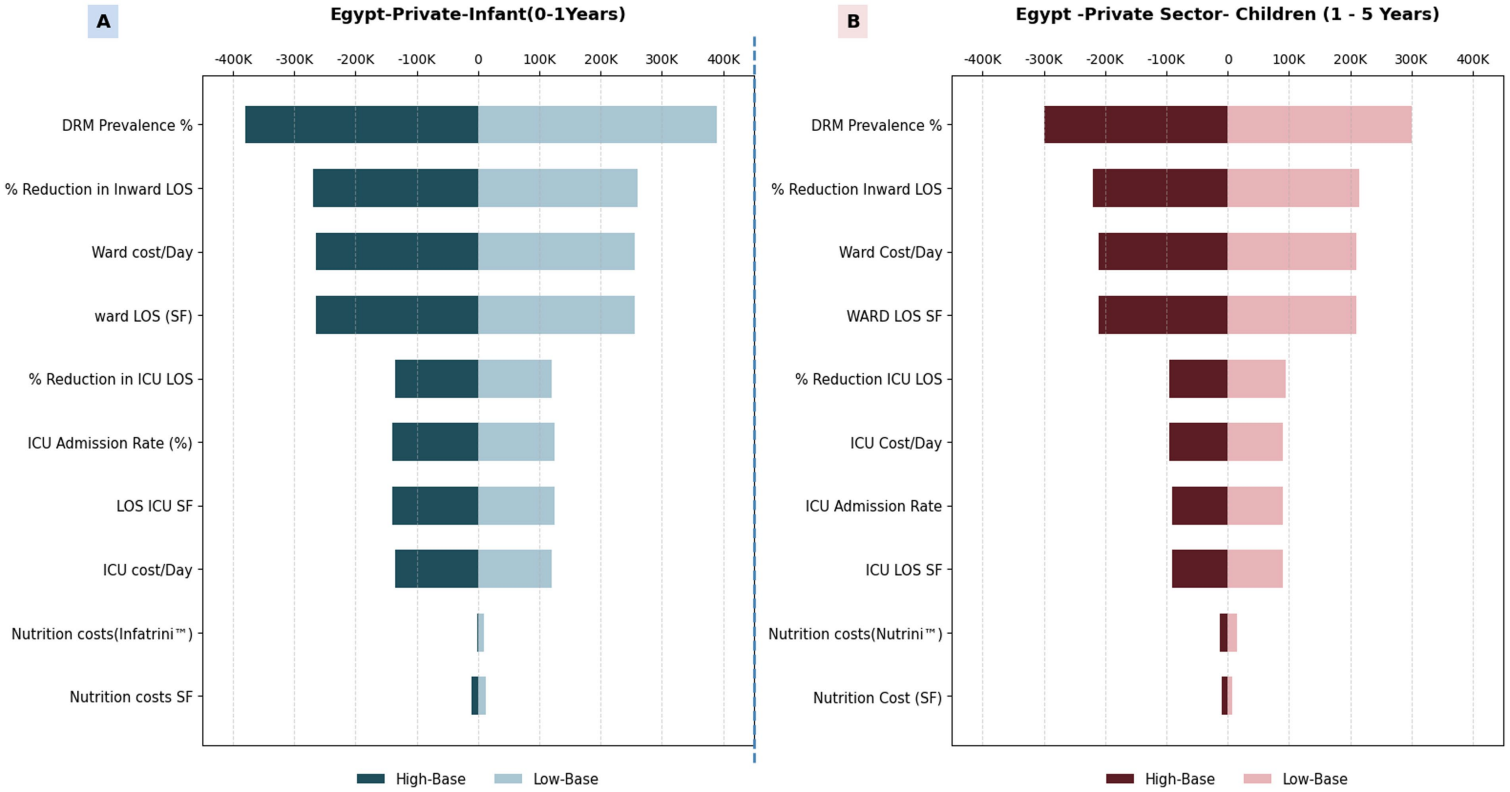
One-way sensitivity analysis tornado diagrams for the private sector. **(A)** The tornado diagram for infants (0–1 years) in Egypt, modeled for Infatrini™ versus standard formula (SF). **(B)** Children (1–5 years) in Egypt, modeled for Nutrini™ versus SF. The cost differences are expressed in Egyptian pounds currency, local currency units. All parameters were varied ±20%. Across all panels, blue shades (dark and light) indicate infants (0–1 years, Infatrini™), while orange shades indicate children (1–5 years, Nutrini™). For each parameter, the darker shade represents the impact on cost savings when the parameter is increased (+20%), while the lighter shade shows the impact when decrease (−20%). Bars represent the impact of varying key parameters on cost savings, with longer bars indicating greater sensitivity.

One-way sensitivity analyses were conducted across all country- and age-specific models to assess the robustness of the cost savings. In all scenarios, cost savings remained positive, confirming the stability of the model under parameter variation. The most influential drivers across settings were the prevalence of pediatric DRM, the percentage reduction in ward LOS, and LOS under the standard formula. Representative tornado diagrams are presented for the public sector (Figures 1A,B) and private sector (Figures 2A,B). The full set of tornado diagrams for all country–setting–age group combinations is provided in the Supplementary Figures 1, 2.

### Scenario analysis results

As shown in Tables 6–9, scenario analyses showed that increasing uptake of Nutrini™ and Infatrini™ was associated with lower total healthcare costs across all countries and healthcare settings. In public hospitals among children aged 1–5 years, Nutrini™ showed increasing cost savings with higher uptake levels (Table 6). In Egypt, savings increased from 10% at 25% uptake to 39% at 100% uptake. Similar findings were observed in Saudi Arabia (11 to 46%), Kuwait (10 to 40%), and the UAE (12 to 49%). Similar trends were also observed in private hospitals across all countries (Table 7).

**Table 6:** Budget impact under different uptake scenarios public hospitals (age 1–5 years).

Country	Uptake % for Nutrini™	Total cost (local currency)	Standard cost 100%	Incremental savings vs. 100% standard	% savings
Egypt	25%	13,129,140.18	14,551,314.82	−1,422,174.64	9.77%
	50%	11,706,965.54		−2,844,349	19.55%
	75%	10,284,790.90		−1,422,174	29.35%
	100%	8,862,616.25		−5,688,698.57	39.09%
KSA	25%	5,311,591.78	5,996,612.38	−685,020	11.42%
	50%	4,626,571.19		−1,370,041	22.85%
	75%	3,941,550.59		−2,055,061	34.27%
	100%	3,256,530.00		−2,740,082.3	45.69%
Kuwait	25%	154,777.62	172,115.98	−17,338	10.07%
	50%	137,439.25		−34,677	20.15%
	75%	120,100.89		−52,015	30.22%
	100%	102,762.53		−69,353.45	40.29%
UAE	25%	107,361,054.14	122,326,694.15	−14,965,640.01	12.23%
	50%	92,331,104.27		−29,995,589	24.52%
	75%	77,429,774.12		−14,965,640	29.32%
	100%	62,464,134.11		−59,862,560.04	48.94%

**Table 7:** Budget impact under different uptake scenarios private hospitals (age 1–5 years).

Country	Uptake % for Nutrini™	Total cost (local currency)	Standard cost 100%	Incremental savings vs. 100% standard	% savings
Egypt	25%	3,647,917.61	4,025,069.05	−377,151.44	9.37%
	50%	3,270,766.17		−754,302.88	18.74%
	75%	2,893,614.73		−1,131,454.32	28.11%
	100%	2,516,463.29		−1,508,605.76	37.48%
KSA	25%	3,102,747.31	3,404,387.98	−301,640.67	8.86%
	50%	2,801,106.63		−603,281.35	17.72%
	75%	2,499,465.96		−904,922.02	26.58%
	100%	2,197,825.29			35.44%
Kuwait	25%	38,408.81	41,094.04	−2,685.23	6.53%
	50%	35,723.57		−5,370.47	13.07%
	75%	33,038.34		−8,055.70	19.60%
	100%	30,353.11		−10,740.93	26.14%
UAE	25%	1,579,565.08	1,698,830.55	−119,265.47	7.02%
	50%	1,460,299.62		−238,530.93	14.04%
	75%	1,341,034.15		−357,796.40	21.06%
	100%	1,221,768.68		−477,061.87	28.08%

**Table 8:** Budget impact under different uptake scenarios public hospitals (age 0–1 years).

Country	Uptake % for Infatrini™	Total cost (local currency)	Standard cost 100%	Incremental savings vs. 100% standard	% savings
Egypt	25%	12,917,789.24	13,606,782.76	−688,993.53	5.06%
	50%	12,228,795.71		−1,377,987.05	10.13%
	75%	11,539,802.19		−2,066,980.58	15.19%
	100%	10,850,808.66		−2,755,974.10	20.25
KSA	25%	−2,755,974.10	5,959,900.22	−678,979.27	11.39%
	50%	4,601,941.67		−1,357,958.55	22.78%
	75%	3,922,962.40		−2,036,937.82	34.18%
	100%	3,243,983.13		−2,715,917.09	45.57%
Kuwait	25%	102,320.23	109,240.98	−6,920.76	6.34%
	50%	95,399.47		−13,841.51	12.67%
	75%	88,478.71		−20,762.27	19.01%
	100%	81,557.96		−27,683.03	25.34
UAE	25%	126,850,787.94	144,899,838.05	−18,049,050.11	12.46%
	50%	108,801,737.83		−36,098,100.22	24.91%
	75%	90,752,687.72		−54,147,150.33	37.37%
	100%	72,703,637.61		−72,196,200.45	49.82%

**Table 9:** Budget impact under different uptake scenarios private hospitals (age 0–1 years).

Country	Uptake % for Infatrini™	Total cost (local currency)	Standard cost 100%	Incremental savings vs. 100% standard	% savings
Egypt	25%	5,081,866.40	5,561,908.20	−480,041.80	8.63%
	50%	4,601,824.60		−960,083.60	17.26%
	75%	4,121,782.79		−1,440,125.41	25.89%
	100%	3,641,740.99		−1,920,167.21	34.52%
KSA	25%	2,515,620.51	2,612,385.55	−96,765.04	3.70%
	50%	2,418,855.47		−193,530.08	7.41%
	75%	2,322,090.44		−290,295.12	11.11%
	100%	2,225,325.40		−387,060.16	14.82%
Kuwait	25%	115,263.07	124,144.15	−8,881.08	7.15%
	50%	106,381.98		−17,762.17	14.31%
	75%	97,500.90		−26,643.25	21.46%
	100%	88,619.82		−35,524.33	28.62%
UAE	25%	314,910.78	342,744.81	−27,834.03	8.12%
	50%	287,076.75		−55,668.06	16.24%
	75%	259,242.72		−83,502.09	24.36%
	100%	231,408.69		−111,336.12	32.48%

Scenario analyses evaluated alternative uptake assumptions for Nutrini™ or Infatrini™ among eligible pediatric DRM patients. Savings were calculated relative to the 100% standard formula baseline scenario. The 100% uptake scenario represents the original base-case assumption and is included for comparison purposes.

For infants aged 0–1 years, Infatrini™ also showed increasing cost savings with higher uptake levels across both public and private hospitals (Tables 8, 9). In public hospitals, savings in Egypt increased from 5% at 25% uptake to 20% at 100% uptake. Similar findings were observed in Saudi Arabia (11 to 46%), Kuwait (6 to 25%), and the UAE (12 to 50%). Similar trends were also observed across private hospital settings.

## Discussion

This budget impact analysis (BIA) demonstrates the clinical and economic value of ENDFs, specifically Infatrini™ and Nutrini™, for hospitalized pediatric patients with DRM across four Middle East countries, which are Egypt, the Kingdom of Saudi Arabia (KSA), Kuwait, and the United Arab Emirates (UAE) in both public and private healthcare settings. Consistent cost reductions were observed in all countries and sectors when these specialized formulas were compared to standard hospital nutrition formulas. These savings were primarily driven by reductions in hospital length of stay (LOS) and associated hospitalization costs, highlighting the close relationship between improved nutritional status, accelerated clinical recovery, and decreased healthcare resource utilization.

The observed LOS reductions with ENDFs are in line with the well-established link between nutritional status and hospital stay duration, where malnutrition is associated with prolonged hospitalization (9, 13). Evidence suggests that timely and appropriate nutritional interventions improve physiological function, enhance treatment response and recovery, ultimately shortening LOS.

In this study, the use of ENDFs resulted in LOS reductions ranged from 19% up to 50% across wards and ICUs in public and private sectors. These real-world findings align with clinical trial data reporting LOS reductions for ENDFS of approximately 29% in wards and 23% in ICUs (14), and are further supported by case studies from India that highlight improved weight gain and recovery in critically ill infants using ENDFS compared to standard formula (17).

The budget impact analysis demonstrated substantial cost savings across all four countries and healthcare sectors with the use of ENDFs, ranging from 15 to 50%. These savings were primarily driven by reductions in hospitalization costs, which accounted for more than 90% of total expenditures in most scenarios, while nutrition costs represented a minimal proportion. Sensitivity analysis confirmed the model’s robustness and identified the pediatric prevalence, % reduction in hospital LOS, and LOS with the standard formula as key drivers of cost savings. Higher DRM prevalence increases the number of patients eligible for intervention, amplifying potential savings. Reductions in LOS and longer LOS directly impact hospitalization costs, with greater LOS reductions yielding larger economic benefits. These findings align with prior budget impact models, including the British Association for Parenteral and Enteral Nutrition (BAPEN) report, which evaluated the economic effects of nutritional interventions in managing malnutrition and similarly identified DRM prevalence and hospital LOS as the most influential parameters affecting cost savings and overall budget impact (20). The scenario analyses showed that both Nutrini™ and Infatrini™ remained cost-saving even at partial uptake levels. Cost savings increased as uptake increased, mainly due to reductions in hospital and ICU length of stay, which are the major drivers of healthcare costs. These findings suggest that introducing energy- and nutrient-dense formulas in pediatric DRM management may reduce healthcare costs even if implementation occurs gradually. Similar findings across countries and healthcare settings support the consistency of the model results.

### The epidemiological insights

While this study primarily estimates the budget impact and cost implications of ENDFs, it also provides valuable real-world insights into the prevalence of disease-related malnutrition (DRM) among hospitalized pediatric patients across the four countries, addressing a critical data gap. The results showed wide variability in DRM prevalence, ranging from 4 to 68% across countries, the two age groups, and healthcare sectors. In public hospitals, prevalence estimates ranged from 21 to 68%, whereas private hospitals consistently reported lower rates between 4 and 13%. These findings are in line with global data, where DRM prevalence in hospitalized children varies between 2.5 and 60% depending on clinical setting, assessment methods, and population characteristics. The highest DRM prevalence was observed in the UAE public sector, reaching 68% among children and 61% among infants-levels and may be comparable to those reported in Latin American hospitals of 70 and 80% prevalence.

The observed differences in DRM prevalence between the public and private sectors can be explained by the higher patient volumes in public hospitals. As presented in Tables 2–5, public facilities serve considerably more pediatric patients annually than private hospitals, managing a broader spectrum of patient characteristics and disease conditions.

The real-world prevalence estimates used in this study align with the limited available regional data. In Egypt, a public hospital study reported an underweight prevalence of 57.8% among hospitalized children with disease-related malnutrition (DRM) (21), comparable to our findings. Similar prevalence rates have been documented in Jordan (19.8%) (22), Saudi Arabia (exceeding 50% among children with cerebral palsy) (22), and Turkey (up to 46.5% in hospitalized children) (23).

## Limitations

First, model inputs relied on expert opinion due to the limited availability of published DRM-epidemiological and economic data across the selected countries. Although local validation was conducted through consultations with key opinion leaders, additional research and epidemiological evidence are needed to strengthen the model’s assumptions. Furthermore, expert elicitation was based on single reported responses at the hospital level, and individual KOL responses were not recorded separately; therefore, measures of variability such as ranges or standard deviations could not be calculated. The elicited estimates may also reflect local clinical practice patterns and expert judgment, which can vary across settings. Second, the analysis used only the hospital LOS for one hospital admission and calculated the percent reduction in LOS as the clinical outcome and a parameter for healthcare resource utilization. Other important parameters, such as readmission rates, % infection rate, and other complications, are not included because of data collection limitations. As a result, the model may under- or overestimate the broader clinical and economic benefits of energy- and nutrient-dense formulas (ENDFs). Additionally, in the Saudi Arabian private sector, a 0 % reduction in ward LOS was reported alongside a 72% reduction in ICU LOS. While this outlier was confirmed through repeated expert validation, it remains unusual and warrants cautious interpretation. Finally, relevant data for the private sector ICU in Kuwait could not be collected, which may affect the accuracy of the results for this setting.

## Conclusion

This study addresses critical global and regional evidence gaps by evaluating the economic impact of ENDFs, specifically Infatrini™ and Nutrini™, as nutritional interventions for managing DRM in pediatric populations. Despite the recognized clinical benefits of such interventions, data on their economic outcomes remain limited globally. In the Middle East, pediatric DRM remains particularly under-recognized, with scarce epidemiological and economic data. To our knowledge, this represents the first multi-country budget impact analysis assessing the introduction of these ENDFs for hospitalized children with DRM across Egypt, Saudi Arabia, Kuwait, and the United Arab Emirates. The findings showed consistent cost savings for ENDFs across the countries and scenarios studied, underscoring the close link between improved nutritional status, faster clinical recovery, and decreased healthcare resource utilization. These findings highlight the urgent need for further research and data generation in this area and support the prioritization of nutrition therapy in pediatric care and health resource allocation.

## Data Availability

The raw data supporting the conclusions of this article will be made available by the authors, without undue reservation.
