# Supplementary material for: Budget impact analysis of energy and nutrient-dense oral nutritional formulas for hospitalized pediatric disease-related malnutrition in the Middle East
Source: Front Nutr. 2026 Jun 15;13:1824690. doi: 10.3389/fnut.2026.1824690 (PMC13310700; doi:10.3389/fnut.2026.1824690)
Supplement: Supplementary file 1 [file Table_1.docx]

**A**

**B**

**D**

**C**

**F**

**E**

**G**

**H**

Figure 1: One Way Sensitivity Analysis -Public Hospitals

**Figure S1. One-Way Sensitivity Analysis Tornado Diagrams for the Public Sector.**
Panel A shows the tornado diagram for infants (0–1 years) in Egypt, modeled for Infatrini™ versus standard Formula (SF). Panel B is for children (1–5 years) in Egypt, modeled for Nutrini™ versus SF. Similarly, Panels C and D correspond to infants and children in Saudi Arabia; Panels E and F to infants and children in Kuwait; and Panels G and H to infants and children in the United Arab Emirates. All Cost differences are expressed in local currency units. All parameters were varied ±20%.
Across all panels, blue shades (dark and light) indicate infants (0–1 years, Infatrini™), while orange shades indicate children (1–5 years, Nutrini™).

For each parameter, the **darker shade** represents the impact on cost savings when the parameter is increased (+20%), while the **lighter shade** shows the impact when decrease (-20%).
Bars represent the impact of varying key parameters on cost savings, with longer bars indicating greater sensitivity.

**A**

**B**

**D**

**C**

**E**

**F**

Figure 2: One Way Sensitivity Analysis -Private Hospitals

**Figure S2. One-Way Sensitivity Analysis Tornado Diagrams for the Private Sector.**
Panel A shows the tornado diagram for infants (0–1 years) in Egypt, modeled for Infatrini™ versus standard Formula (SF). Panel B is for children (1–5 years) in Egypt, modeled for Nutrini™ versus SF. Similarly, Panels C and D correspond to infants and children in Saudi Arabia; Panels E and F to infants and children in the United Arab Emirates. All Cost differences are expressed in local currency units. All parameters were varied ±20%.
Across all panels, blue shades (dark and light) indicate infants (0–1 years, Infatrini™), while orange shades indicate children (1–5 years, Nutrini™).

For each parameter, the **darker shade** represents the impact on cost savings when the parameter is increased (+20%), while the **lighter shade** shows the impact when decrease (-20%).
Bars represent the impact of varying key parameters on cost savings, with longer bars indicating greater sensitivity.
